# Foot-and-Mouth Disease Virus 3A Protein Causes Upregulation of Autophagy-Related Protein LRRC25 To Inhibit the G3BP1-Mediated RIG-Like Helicase-Signaling Pathway

**DOI:** 10.1128/JVI.02086-19

**Published:** 2020-03-31

**Authors:** Wenping Yang, Dan Li, Yi Ru, Juncui Bai, Jingjing Ren, Jing Zhang, Lulu Li, Xiangtao Liu, Haixue Zheng

**Affiliations:** a State Key Laboratory of Veterinary Etiological Biology, Lanzhou Veterinary Research Institute, Chinese Academy of Agricultural Sciences, Lanzhou, Gansu, China; b OIE/National Foot and Mouth Disease Reference Laboratory, Lanzhou Veterinary Research Institute, Chinese Academy of Agricultural Sciences, Lanzhou, Gansu, China; Hudson Institute of Medical Research

**Keywords:** 3A, FMDV, G3BP1, LRRC25, autophagy

## Abstract

Foot-and-mouth disease virus (FMDV) is one of the most notorious pathogens in the global livestock industry. To establish an infection, FMDV needs to counteract host antiviral responses. Several studies have shown how FMDV suppresses the type I interferon (IFN) response; however, whether FMDV modulates the integrated autophagy and innate immunity remains largely unknown. Here, the porcine Ras-GAP SH3-binding protein 1 (G3BP1) was shown to promote the retinoic acid-inducible gene I (RIG-I)-like helicase (RLH) signaling by upregulating the expression of RIG-I and melanoma differentiation-associated gene 5 (MDA5). FMDV nonstructural protein 3A interacted with G3BP1 to inhibit G3BP1 expression and G3BP1-mediated RLH signaling by upregulating the expression of autophagy-related protein LRRC25. In addition, 3A proteins of other picornaviruses, including Seneca Valley virus (SVV) 3A, enterovirus 71 (EV71) 3A, and encephalomyocarditis virus (EMCV) 3A, also showed similar actions. Taking the data together, we elucidated, for the first time, a novel mechanism by which FMDV has evolved to inhibit IFN signaling and counteract host innate antiviral responses by autophagy.

**IMPORTANCE** We show that foot-and-mouth disease virus (FMDV) 3A inhibits retinoic acid-inducible gene I (RIG-I)-like helicase signaling by degrading G3BP1 protein. Furthermore, FMDV 3A reduces G3BP1 by upregulating the expression of autophagy-related protein LRRC25. Additionally, other picornavirus 3A proteins, such as Seneca Valley virus (SVV) 3A, enterovirus 71 (EV71) 3A, and encephalomyocarditis virus (EMCV) 3A, also degrade G3BP1 by upregulating LRRC25 expression. This study will help us improve the design of current vaccines and aid the development of novel control strategies to combat FMD.

## Abstract (precis)

We show that foot-and-mouth disease virus (FMDV) 3A inhibits retinoic acid-inducible gene I (RIG-I)-like helicase signaling by degrading G3BP1 protein. Furthermore, FMDV 3A reduces G3BP1 by upregulating the expression of autophagy-related protein LRRC25. Additionally, other picornavirus 3A proteins, such as Seneca Valley virus (SVV) 3A, enterovirus 71 (EV71) 3A, and encephalomyocarditis virus (EMCV) 3A, also degrade G3BP1 by upregulating LRRC25 expression. This study will help us improve the design of current vaccines and aid the development of novel control strategies to combat FMD.

## INTRODUCTION

Foot-and-mouth disease (FMD) is one of the most important viral diseases of livestock and causes significant economic losses to the pig industry (1). The etiologic agent FMD virus (FMDV) is a member of the family *Picornaviridae* and genus *Aphthovirus* (2). FMDV has a single-stranded, positive-sense RNA genome of approximately 8.5 kb and encodes a large polyprotein that is processed into four structural proteins (VP1 to -4) and eight nonstructural proteins (L^pro^, 2A, 2B, 2C, 3A, 3B, 3C^pro^, and 3D) by three virus-encoded proteinases (L^pro^, 2A, and 3C^pro^) (1, 3, 4). To rapidly and efficiently replicate in the host, many FMDV proteins have evolved a range of strategies to antagonize and escape the innate immune response (4). For example, the structural and nonstructural proteins such as VP3 (3, 5), VP1 (6), 3A (7), 3C^pro^ (4, 8), and L^pro^ (9, 10) modulate the innate immune response through distinct mechanisms.

The innate immune response is pivotal for host defense against viral infection. Upon infection, viral RNA is detected by cytosolic sensors. In most cell types, the cytoplasmic retinoic acid-inducible gene I (RIG-I)-like receptors (RLRs), including RIG-I and melanoma differentiation-associated gene 5 (MDA5), play key roles in sensing RNA virus invasion (11, 12). RIG-I and MDA5, which are composed of two caspase recruitment domains (CARDs) and an RNA helicase domain, have been shown to participate in antiviral innate immunity (13).

Signaling mediated by RIG-I and MDA5 is delicately controlled by many host proteins. For example, hemoglobin subunit beta (HB) directly inhibits MDA5-mediated signaling by reducing MDA5-double-stranded RNA (dsRNA) affinity while promoting the RIG-I-mediated signaling through enhancing K63-linked RIG-I ubiquitination (14); knockdown of insulin-like growth factor 1 receptor (IGF-1R) triggers viral RNA sensor MDA5- and RIG-I-mediated mitochondrial apoptosis in cancer cells (15). Tripartite motif 38 (TRIM38) positively regulates MDA5- and RIG-I-mediated induction of downstream genes and acts as a small ubiquitin-like modifier (SUMO) E3 ligase for their dynamic sumoylation (16). Zinc finger CCHC domain-containing protein 3 (ZCCHC3) binds to dsRNA and enhances the binding of RIG-I and MDA5 to dsRNA. ZCCHC3 also recruits the E3 ubiquitin ligase TRIM25 to RIG-I and MDA5 complexes, which facilitates RIG-I and MDA5 K63-linked polyubiquitination and subsequent activation. (17). It has been reported that Ras-GAP SH3-binding protein 1 (G3BP1) is localized with RIG-I (18). Therefore, in this study, we investigated whether porcine G3BP1 mediates the RLH signaling pathway and how FMDV proteins regulate the G3BP1-mediated signaling pathway.

G3BP1, also known as G3BP or HDH-VIII, is a ubiquitously expressed protein and functions as a sequence-specific, phosphorylation-dependent helicase, a cofactor, an endoribonuclease, and more (19). Recently, it has been reported that FMDV L^pro^ and 3C^pro^ may cleave G3BP1 to inhibit stress granule (SG) formation; however, FMDV L^pro^ and 3C^pro^ do not interact with G3BP1 (20, 21). This finding prompted us to determine the FMDV proteins that interact with G3BP1 and the mechanism by which it regulates G3BP1 to facilitate FMDV replication and growth. Here, we found that FMDV 3A interacted with G3BP1 and inhibited the G3BP1-mediated RLH signaling pathway. In addition, FMDV 3A degraded G3BP1 by upregulating autophagy-related protein LRRC25 to inhibit the RLH signaling pathway, which, in turn, increased FMDV replication and growth. These findings reveal a mechanism of critical importance that allows FMDV to evade the immune system.

## RESULTS

### G3BP1 is involved in the defense response against FMDV.

**FIG 1:**
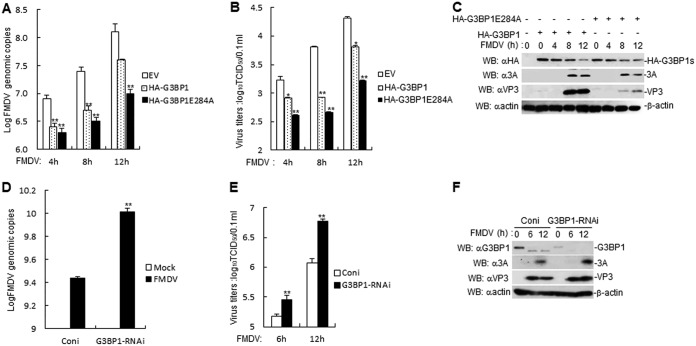
G3BP1 and G3BP1E284A inhibit FMDV genomic copies and titer. (A) Effects of overexpression of G3BP1 and G3BP1E284A on FMDV replication in PK-15 cells. G3BP1 and G3BP1E284A overexpression in PK-15 cells were infected with FMDV (MOI, 1.0) for the indicated times. FMDV genome replication was evaluated by RT-PCR. The experiment shown is a representative experiment of three independent experiments with mean ± SD of three technical replicates. (B) Effects of overexpression of G3BP1 and G3BP1E284A on FMDV titers in PK-15 cells. G3BP1 and G3BP1E284A overexpression in PK-15 cells were infected with FMDV (MOI, 1.0) for the indicated times. The virus titers in the supernatants collected were determined by immunofluorescence assay and expressed as TCID_50_/0.1 ml. The experiment shown is a representative experiment of three independent experiments with mean ± SD of three technical replicates. (C) FMDV inhibits the expression of G3BP1 and G3BP1E284A proteins. G3BP1 and G3BP1E284A overexpression in PK-15 cells were infected with FMDV (MOI, 1.0) for the indicated times. G3BP1, G3BP1E284A, FMDV 3A, and FMDV VP3 proteins were detected by immunoblotting analysis with indicated antibodies. (D) Effects of G3BP1-RNAi on FMDV replication in PK-15 cells. G3BP1 knockdown in PK-15 cells were infected with FMDV (MOI, 1.0) for the indicated times. FMDV genome replication was evaluated by RT-PCR. The experiment shown is a representative experiment of three independent experiments with mean ± SD of three technical replicates. (E) Effects of G3BP1-RNAi on FMDV titers in PK-15 cells. G3BP1 knockdown in PK-15 cells were infected with FMDV (MOI, 1.0) for the indicated times. The virus titers in the supernatants collected were determined by immunofluorescence assay and expressed as TCID_50_/0.1 ml. The experiment shown is a representative experiment of three independent experiments with mean ± SD of three technical replicates. (F) Effects of G3BP1-RNAi on FMDV proteins in PK-15 cells. G3BP1 knockdown in PK-15 cells were infected with FMDV (MOI, 1.0) for the indicated times. G3BP1, FMDV 3A, and FMDV VP3 proteins were detected by immunoblotting analysis with indicated antibodies. SD, standard deviation; G3BP1s, G3BP1 and G3BP1E284A; EV, empty vector; Coni, control-RNAi.

Assuming that FMDV L^pro^ and 3C^pro^ cleave G3BP1 to inhibit SG formation (20, 21), we investigated the effects of G3BP1 on FMDV replication and growth. Previous studies have shown that FMDV 3C^pro^ cleaves G3BP1 at glutamic acid-284 (E284) (21). G3BP1 or G3BP1E284A overexpression inhibits FMDV genomic copies and titer in porcine kidney 15 (PK-15) cells, but G3BP1E284A is more potent than G3BP1 in inhibiting FMDV genomic copies and titer (Fig. 1A and B). Furthermore, FMDV infection inhibited G3BP1 and G3BP1E284A expression (Fig. 1C), suggesting that other FMDV proteins mediated G3BP1 expression inhibition, other than FMDV 3C^pro^. Next, the function of endogenous G3BP1 in FMDV genomic copies and titer was investigated. An RNA interference (RNAi) plasmid was constructed for G3BP1, and RNAi plasmids markedly reduced the expression of endogenous G3BP1 in PK-15 cells (Fig. 1F). In reverse transcriptase PCR (RT-PCR) and titer experiments, FMDV genomic copies and titer were increased in G3BP1-knockdown PK-15 cells (Fig. 1D and E). In addition, there was an increased expression of 3A and VP3 in G3BP1-knockdown PK-15 cells compared with control cells (Fig. 1F). Collectively, these results suggest that G3BP1 negatively regulates FMDV replication and growth.

### Porcine G3BP1 promotes the RLH-mediated signaling pathway.

**FIG 2:**
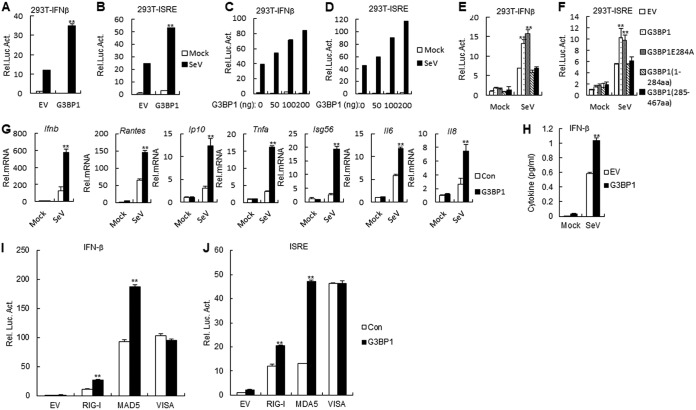
Porcine G3BP1 inhibits the RLH signaling pathway upstream of VISA. (A and B) Effects of overexpression of porcine G3BP1 on SeV-triggered IFN-β promoter (A) and ISRE (B) activation. HEK293T cells (1 × 10^5^) were transfected with the IFN-β reporter or ISRE (0.1 μg) and the indicated expression (0.1 μg) plasmids. Twenty hours after transfection, cells were infected with SeV or left uninfected for 12 h before luciferase assays were performed. The experiment shown is a representative experiment of three independent experiments with mean ± SD of three technical replicates. EV, empty vector. (C and D) Dose-dependent effects of porcine G3BP1 on SeV-triggered activation of IFN-β promoter and ISRE. The experiments were similarly performed as in A. The experiment shown is a representative experiment of three independent experiments with mean ± SD of three technical replicates. (E and F) Effects of G3BP1 and its mutants on SeV-triggered IFN-β promoter and ISRE activation. The experiments were similarly performed as in A. The experiment shown is a representative experiment of three independent experiments with mean ± SD of three technical replicates. (G) G3BP1 inhibits SeV-triggered induction of downstream antiviral genes. HEK293T cells were transfected with the indicated expression plasmids for 20 h and then infected with SeV or left untreated for 8 h before RT-PCR was performed. The experiment shown is a representative experiment of three independent experiments with mean ± SD of three technical replicates. (H) Effects of G3BP1 overexpressed on secretion of IFN-β induced by SeV in HEK293T cells. G3BP1-overexpressed HEK293T cells were infected with SeV for 12 h. The culture medium was collected for quantization of the indicated cytokines by ELISA. The experiment shown is representative experiments of three independent experiments with mean ± SD of three technical replicates. (I and J) Effects of G3BP1 on IFN-β promoter (I) and ISRE (J) activation by various signaling components. HEK293T cells (1 × 10^5^) were transfected with IFN-β promoter or ISRE reporter (0.1 μg), and expression plasmids for G3BP1 and the indicated proteins (0.1 μg each). Luciferase assays were performed 24 h after transfection. The experiment shown is a representative experiment of three independent experiments with mean ± SD of three technical replicates. EV, empty vector.

G3BP1 has been shown to bind to viral dsRNA and RIG-I to enhance interferon-β (IFN-β) responses (18). It has also been shown to share 92% sequence identity with its porcine ortholog, from which observation suspicion of porcine G3BP1 participation in RIG-I-mediated signaling arose. In reporter assays, overexpression of porcine G3BP1 increased Sendai virus (SeV)-triggered activation of the IFN-β promoter and interferon-stimulated response element (ISRE) in human embryonic kidney 293T (HEK293T) cells (Fig. 2A and B). Further experiments indicated that overexpression of G3BP1 inhibited SeV-triggered activation of the IFN-β promoter and ISRE in a dose-dependent manner in HEK293T cells (Fig. 2C and D). In addition, we also found that G3BP1 and G3BP1E284A increased the SeV-induced IFN-β promoter and ISRE activation, not including G3BP1 (1 to 284 amino acid [aa]) and G3BP1 (285 to 467 aa) (Fig. 2E and F). In an RT-PCR experiment, we observed that SeV-triggered transcription of the *Ifnb1*, *Rantes*, *Ip10*, *Tnfa*, *Isg56*, *Il6*, and *Il8* genes were increased in G3BP1-overexpressed HEK293T cells compared with control cells (Fig. 2G). Additionally, enzyme-linked immunosorbent assay (ELISA) experiments indicated that the levels of secreted IFN-β induced by SeV infection increased in G3BP1-overexpressed HEK293T cells compared with wild-type cells (Fig. 2H). These results suggest that porcine G3BP1 also positively regulates a virus-triggered signaling pathway.

Various components are involved in virus-triggered signaling pathways. As shown in Fig. 2I and J, G3BP1 potentiates the IFN-β promoter and ISRE activation, mediated by RIG-I and MDA5 but not virus-induced signaling adapter (VISA). These results suggest that G3BP1 targets RIG-I and MDA5.

### FMDV 3A interacts with G3BP1.

**FIG 3:**
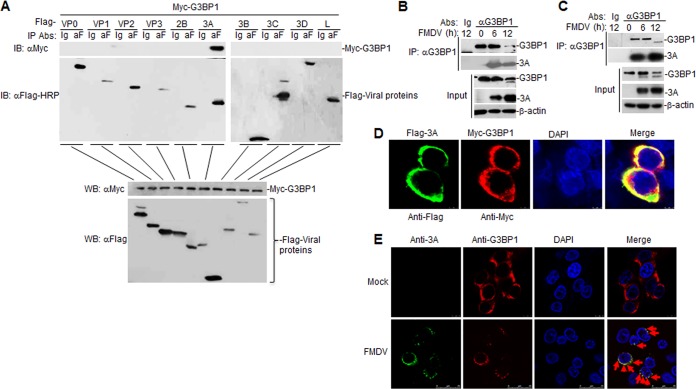
(A) The G3BP1 protein interacts with the FMDV 3A protein. HEK293T cells were transfected with the indicated plasmids for 20 h before coimmunoprecipitation and immunoblotting analysis with the indicated antibodies. (B and C) Endogenous G3BP1 is associated with FMDV 3A in PK-15 and PAM cells. PK-15 (B) or PAM cells (C) were infected with FMDV (MOI, 1.0) for the indicated times. Coimmunoprecipitation and immunoblotting analysis were performed with the indicated antibodies. (D) Colocalization of FMDV 3A protein with G3BP1. HEK293T cells were cotransfected with Flag-3A and Myc-G3BP1. One day after transfection, the cells were fixed and subjected to indirect immunofluorescence to detect Flag-3A (green) and Myc-G3BP1 (red) with mouse anti-Myc and rabbit anti-Flag antibodies. The position of the nucleus is indicated by 4′,6-diamidino-2-phenylindole (DAPI; blue) staining in the merged image. (E) Colocalization of FMDV 3A protein with endogenous G3BP1. PK-15 cells were mock infected or infected with FMDV. The cells were fixed at 12 h postinfection and subjected to indirect immunofluorescence to detect FMDV 3A protein (green) and G3BP1 (red) with mouse anti-3A protein and rabbit anti-G3BP1 antibodies. Red arrows indicate colocations. The position of the nucleus is indicated by DAPI (blue) staining in the merged image. EV, empty vector.

Next, the FMDV proteins that interact with G3BP1 were determined. In transient-transfection and coimmunoprecipitation experiments, G3BP1 interacted with FMDV 3A but not VP0, VP1, VP2, VP3, 2B, 3B, 3C^pro^, 3D, and L^pro^ proteins (Fig. 3A). Endogenous coimmunoprecipitation experiments indicated that FMDV 3A was associated with G3BP1 in PK-15 cells and porcine alveolar macrophages (PAMs) following FMDV infection (Fig. 3B and C). To examine the colocalization of the FMDV 3A protein with G3BP1, HEK293T cells were cotransfected with plasmids expressing Flag-3A and Myc-G3BP1, and the subcellular localization of 3A protein and G3BP1 was examined by confocal microscopy (Fig. 3D). To confirm that endogenous G3BP1 colocalizes with the 3A protein, PK-15 cells were infected with FMDV and analyzed by confocal microscopy. Confocal images of the cells immunostained with anti-3A and anti-G3BP1 antibodies showed colocalization of G3BP1 with the FMDV 3A protein (Fig. 3E). Collectively, these findings confirm that FMDV 3A interacts with G3BP1.

### FMDV 3A enhances FMDV genomic copies and titer.

**FIG 4:**
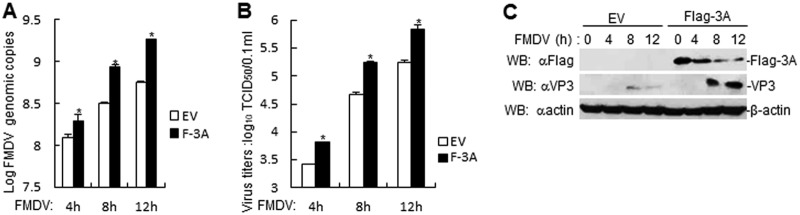
FMDV 3A increases FMDV genomic copies and titer. (A) Effects of FMDV 3A overexpression in PK-15 cells on FMDV replication. FMDV 3A overexpression in PK-15 cells were infected with FMDV (MOI, 1.0) for the indicated times. FMDV genome replication was evaluated by RT-PCR. The experiment shown is a representative experiment of three independent experiments with mean ± SD of three technical replicates. (B) Effects of FMDV 3A overexpression on FMDV titers in PK-15 cells. FMDV 3A overexpression in PK-15 cells were infected with FMDV (MOI, 1.0) for the indicated times. The virus titers in the supernatants collected were determined by immunofluorescence assay and expressed as TCID_50_/0.1 ml. The experiment shown is a representative experiment of three independent experiments with mean ± SD of three technical replicates. (C) Effects of FMDV 3A overexpression on FMDV proteins in PK-15 cells. FMDV 3A overexpression in PK-15 cells were infected with FMDV (MOI, 1.0) for the indicated times. Flag-3A and FMDV VP3 proteins were detected by immunoblotting analysis with indicated antibodies.

Next, the effect of FMDV 3A on FMDV genomic copies and titer was examined. RT-PCR and titer experiments demonstrated that the FMDV 3A-overexpressed PK-15 cells increased FMDV genomic copies and titer (Fig. 4A and B). In accordance with this finding, FMDV 3A-overexpressed PK-15 cells also increased FMDV VP3 expression (Fig. 4C). These results suggest that FMDV 3A promotes FMDV replication and growth.

### FMDV 3A represses the G3BP1-mediated RLH antiviral signaling.

**FIG 5:**
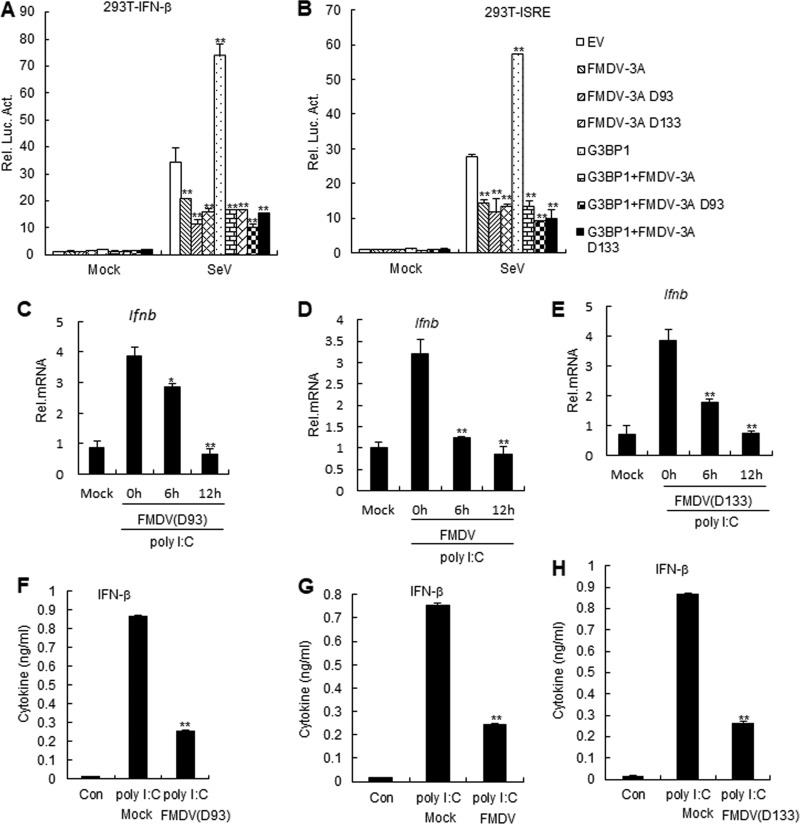
FMDV 3A inhibits G3BP1-mediated RLH signaling pathway. (A and B) Effects of FMDV 3A and its mutants on G3BP1-mediated RLH signaling pathway during SeV infection. HEK293T cells (1 × 10^5^) were transfected with the IFN-β reporter or ISRE (0.1 μg) and the indicated expression (0.1 μg) plasmids. Twenty hours after transfection, cells were infected with SeV or left uninfected for 12 h before luciferase assays were performed. The experiment shown is a representative experiment of three independent experiments with mean ± SD of three technical replicates. EV, empty vector. (C, D, and E) Effects of FMDV and its mutants on poly(I·C)-induced signaling pathway in PK-15 cells. PK-15 cells were transfected with poly(I·C) (1 μg/ml) for 18 h. Cells were left uninfected or infected with FMDV or its mutants (MOI, 1.0) for 12 h, and RT-PCR experiments were performed. The experiment shown is a representative experiment of three independent experiments with mean ± SD of three technical replicates. (F, G, and H) Effects of FMDV and its mutants on secretion of IFN-β induced by poly(I·C) in PAM cells. The experiments were similarly performed as in C. The experiment shown is a representative experiment of three independent experiments with mean ± SD of three technical replicates. EV, empty vector; Con, control.

Previous studies have shown that amino acid deletion at positions 93 to 102 in FMDV 3A (hereafter referred to as 3A D93) and 133 to 143 in FMDV 3A (hereafter referred to as 3A D133) could alter the host range of FMDV (22). In addition, in light of the results of our previous experiments, we investigated the effect of FMDV 3A on the G3BP1-mediated RLH signaling pathway. This prompted further investigation to clarify whether FMDV 3A and its mutants affected RLH signaling or G3BP1-mediated RLH signaling. In reporter assays, FMDV 3A, 3A D93, and 3A D133 inhibited SeV-triggered activation of the IFN-β promoter and ISRE (Fig. 5A and B). To further confirm the effect of 3A mutants on RLH signaling, PK-15 cells were infected with wild-type FMDV and deletion mutant FMDV. The results indicated that wild-type FMDV and deletion mutant FMDV (3A D93 and 3A D133) inhibited poly(I·C)-triggered IFN-β mRNA levels in PK-15 cells (Fig. 5C and D and E). Additionally, wild-type FMDV and deletion mutant FMDV (3A D93 and 3A D133) inhibited poly(I·C)-triggered IFN-β expression by ELISAs in PAM cells compared to control cells (Fig. 5F to H). Collectively, these results suggest that FMDV 3A inhibits G3BP1-mediated RLH signaling.

### FMDV 3A degrades G3BP1 via autophagy.

**FIG 6:**
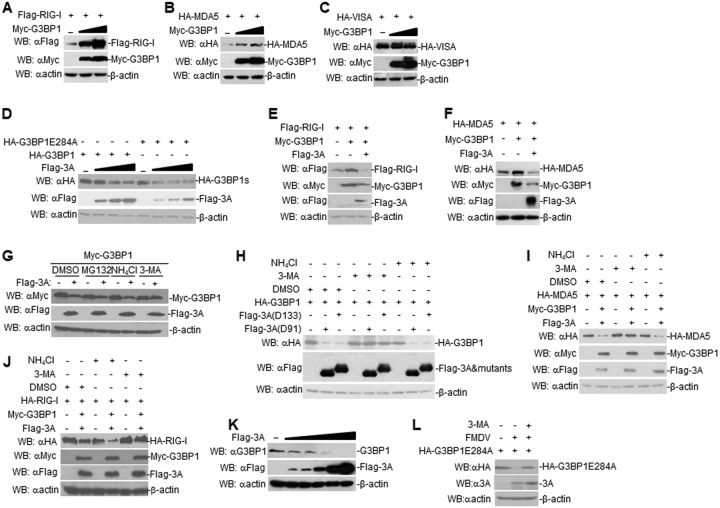
FMDV 3A degrades G3BP1 by autophagy. (A) Dose-dependent effects of G3BP1 on the expression of RIG-I in HEK293T cells. HEK293T cells (2 × 10^5^) were transfected with the Flag-RIG-I (0.5 μg) and Myc-G3BP1 plasmids (0 μg, 0.1 μg, or 0.2 μg) for 24 h. The cell lysates were analyzed by immunoblotting with anti-Flag, anti-β-actin, or anti-Myc antibodies. (B) Dose-dependent effects of G3BP1 on the expression of MDA5 in HEK293T cells. The experiments were similarly performed as in A. (C) Dose-dependent effects of G3BP1 on the expression of VISA in HEK293T cells. The experiments were similarly performed as in A. (D) Dose-dependent effects of FMDV 3A on the expression of G3BP1 and G3BP1E284A in HEK293T cells. The experiments were similarly performed as in A. G3BP1s, G3BP1 and G3BP1E284A. (E) Effects of G3BP1 with or without FMDA 3A on the expression of RIG-I in HEK293T cells. HEK293T cells were transfected with indicated plasmids for 24 h. The cell lysates were analyzed by immunoblotting with anti-Flag, anti-β-actin, or anti-Myc antibodies. (F) Effects of G3BP1 with or without FMDA 3A on the expression of MDA5 in HEK293T cells. The experiments were similarly performed as in E. (G) FMDV 3A mediates the autophagy pathway degradation of G3BP1. HEK293T cells (2 × 10^5^) were transfected with the indicated plasmids. Eighteen hours after transfection, the cells were treated with the indicated inhibitors (MG132 [25 μM], 3-MA [0.5 μg/μl], and NH_4_Cl [25 mM]) for 6 h before immunoblotting analysis. (H) FMDV 3A mutants mediate the autophagy pathway degradation of G3BP1. The experiments were similarly performed as in G. (I) Effects of FMDV 3A and G3BP1 on the expression of MDA5 by autophagy pathway. The experiments were similarly performed as in G. (J) Effects of FMDV 3A and G3BP1 on the expression of RIG-I by autophagy pathway. The experiments were similarly performed as in G. (K) Effects of FMDV 3A on endogenous G3BP1. PK-15 cells were transfected with FMDV 3A for 24 h. The cell lysates were analyzed by immunoblotting. (L) Effects of FMDV on the expression of G3BP1E284A by autophagy pathway. G3BP1E284A overexpression in PK-15 cells were infected with FMDV or left uninfected for 4 h. Cells were treated or untreated with 3-MA for 6 h. The cell lysates were analyzed by immunoblotting.

Next, regulation of RLH signaling by porcine G3BP1 was investigated. In transient-transfection experiments, G3BP1 increased RIG-I and MDA5 expression but not VISA expression in a dose-dependent manner in HEK293T cells (Fig. 6A to C). Furthermore, we found that FMDV 3A degraded G3BP1 and G3BP1E284A in a dose-dependent manner in HEK293T cells (Fig. 6D). Moreover, we also observed that FMDV 3A inhibited the expression of G3BP1-mediated RIG-I and MDA5 (Fig. 6E and F). These data collectively demonstrate that FMDV 3A degrades G3BP1.

To investigate the mechanisms responsible for the role of FMDV 3A on the stability of G3BP1, G3BP1-expressed or G3BP1 and FMDV 3A-coexpressed HEK293T cells were treated with various inhibitors for protein degradation pathways. 3-Methyladenine (3-MA), an autophagosome inhibitor, but not the lysosome inhibitor ammonium chloride (NH_4_Cl) or proteasome inhibitor MG132, markedly inhibited the degradation of G3BP1 in G3BP1 and FMDV 3A-coexpressed HEK293T cells (Fig. 6G). Consistently, 3-MA, but not NH_4_Cl, inhibited the degradation of G3BP1 in G3BP1 and FMDV 3A mutant-coexpressed HEK293T cells (Fig. 6H). Furthermore, 3-MA, but not NH_4_Cl, was found to block FMDV 3A- and G3BP1-mediated MDA5 and RIG-I expression (Fig. 6I and J). Additionally, FMDV 3A degraded endogenous G3BP1 in PK-15 cells (Fig. 6K). Consistently, 3-MA also blocked FMDV infection-mediated G3BP1E284A degradation in PK-15 cells (Fig. 6L). These results suggest that FMDV 3A degrades G3BP1 by the autophagy pathway.

### FMDV 3A degrades G3BP1 through upregulating LRRC25 expression.

**FIG 7:**
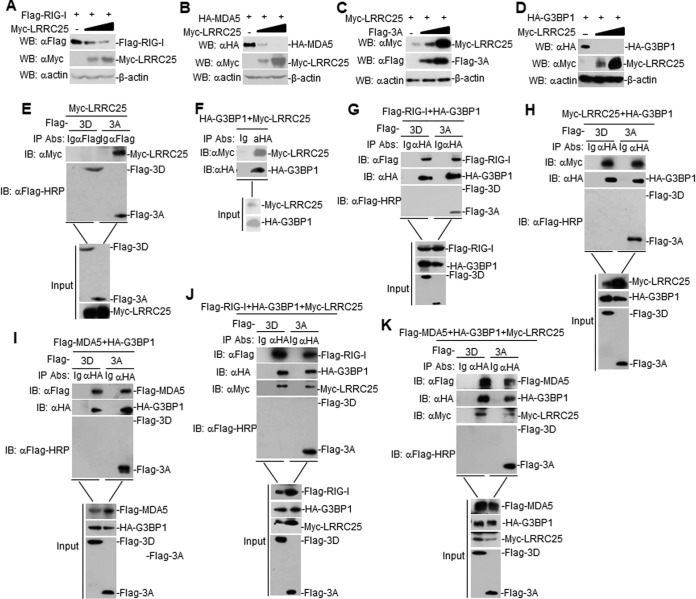
FMDV 3A degrades G3BP1 by the autophagy-related protein LRRC25. (A) Dose-dependent effects of LRRC25 on the expression of RIG-I in HEK293T cells. HEK293T cells (2 × 10^5^) were transfected with the Flag-RIG-I (0.5 μg) and Myc-LRRC25 plasmids (0 μg, 0.1 μg, or 0.2 μg) for 24 h. The cell lysates were analyzed by immunoblotting with anti-Flag, anti-β-actin, or anti-Myc antibodies. (B) Dose-dependent effects of LRRC25 on the expression of MDA5 in HEK293T cells. The experiments were similarly performed as in A. (C) Dose-dependent effects of FMDV 3A on the expression of LRRC25 in HEK293T cells. The experiments were similarly performed as in A. (D) Dose-dependent effects of LRRC25 on the expression of G3BP1 in HEK293T cells. The experiments were similarly performed as in A. (E) Interaction between FMDV 3A or 3D and LRRC25 in mammalian overexpression system. HEK293T cells were transfected with the indicated plasmids for 24 h. Coimmunoprecipitation and immunoblotting analysis were performed with the indicated antibodies. (F) Interaction between G3BP1 and LRRC25 in mammalian overexpression system. The experiments were similarly performed as in E. (G and H) Interaction between G3BP1, FMDV 3A or 3D, and LRRC25 or RIG-I in HEK293T cells. The experiments were similarly performed as in E. (I) Interaction between G3BP1, FMDV 3A or 3D, and MDA5 in HEK293T cells. The experiments were similarly performed as in E. (J and K) Interaction between G3BP1, FMDV 3A or 3D, LRRC25, and MDA5 or RIG-I in HEK293T cells. The experiments were similarly performed as in E.

It has been reported that leucine rich repeat-containing 25 (LRRC25) inhibits IFN-β signaling by targeting RIG-I for autophagic degradation (23). This finding prompted us to investigate whether LRRC25 is involved in FMDV 3A- and G3BP1-mediated RLH signaling. In transient-transfection experiments, we found that LRRC25 inhibited RIG-I and MDA5 expression in a dose-dependent manner in HEK293T cells (Fig. 7A and B). Furthermore, FMDV 3A was found to enhance LRRC25 expression in a dose-dependent manner in HEK293T cells (Fig. 7C). In addition, LRRC25 inhibited the expression of G3BP1 in HEK293T cells (Fig. 7D). In transient-transfection and coimmunoprecipitation experiments, FMDV 3A and G3BP1 were shown to interact with LRRC25 in HEK293T cells, but FMDV 3D and LRRC25 showed no interaction (Fig. 7E and F). Furthermore, FMDV 3A, but not 3D, G3BP1, LRRC25, RIG-I, and MDA5 formed a complex (Fig. 7G to K). Taken together, these results suggest that FMDV 3A degrades G3BP1 by upregulating LRRC25 expression.

### LRRC25 increases FMDV genomic copies and titer.

**FIG 8:**
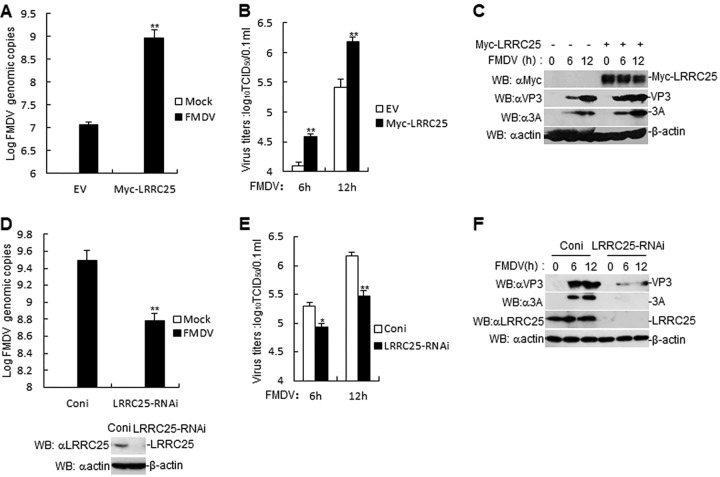
LRRC25 increases FMDV genomic copies and titer. (A) Effects of LRRC25 overexpression on FMDV replication in PK-15 cells. LRRC25 overexpression in PK-15 cells were infected with FMDV (MOI, 1.0) for the indicated times. FMDV genome replication was evaluated by RT-PCR. The experiment shown is a representative experiment of three independent experiments with mean ± SD of three technical replicates. (B) Effects of overexpression of LRRC25 on FMDV titers in PK-15 cells. LRRC25 overexpression in PK-15 cells were infected with FMDV (MOI, 1.0) for the indicated times. The virus titers in the supernatants collected were determined by immunofluorescence assay and expressed as TCID_50_/0.1 ml. The experiment shown is a representative experiment of three independent experiments with mean ± SD of three technical replicates. (C) Effects of overexpression of LRRC25 on FMDV proteins in PK-15 cells. LRRC25 overexpression in PK-15 cells were infected with FMDV (MOI, 1.0) for the indicated times. Myc-LRRC25, FMDV 3A, and FMDV VP3 proteins were detected by immunoblotting analysis with the indicated antibodies. (D) Effects of LRRC25-RNAi on FMDV replication in PK-15 cells. LRRC25 knockdown in PK-15 cells were infected with FMDV (MOI, 1.0) for the indicated times. FMDV genome replication was evaluated by RT-PCR. The experiment shown is a representative experiment of three independent experiments with mean ± SD of three technical replicates. The bottom blots show the expression levels of endogenous LRRC25 as detected by anti-LRRC25 antibody. (E) Effects of LRRC25-RNAi on FMDV titers in PK-15 cells. LRRC25 knockdown in PK-15 cells were infected with FMDV (MOI, 1.0) for the indicated times. The virus titers in the supernatants collected were determined by immunofluorescence assay and expressed as TCID_50_/0.1 ml. The experiment shown is a representative experiment of three independent experiments with mean ± SD of three technical replicates. (F) Effects of LRRC25-RNAi on FMDV proteins in PK-15 cells. LRRC25 knockdown in PK-15 cells were infected with FMDV (MOI, 1.0) for the indicated times. LRRC25, FMDV 3A, and FMDV VP3 proteins were detected by immunoblotting analysis with indicated antibodies. SD, standard deviation; EV, empty vector.

We next determined whether endogenous LRRC25 regulates FMDV genomic copies and titer. RT-PCR and titer experiments revealed that LRRC25-overexpressed PK-15 cells increased FMDV genomic copies and titer (Fig. 8A and B). Consistently, the expression of FMDV 3A and VP3 proteins were increased in LRRC25-overexpressed PK-15 cells (Fig. 8C). A porcine LRRC25-RNAi plasmid was constructed, and immunoblotting analysis indicated that it markedly inhibited the expression of endogenous LRRC25 in PK-15 cells (Fig. 8D). In RT-PCR and titer experiments, we observed that FMDV genomic copies and titer were reduced in LRRC25-knockdown PK-15 cells compared with control cells (Fig. 8D and E). Furthermore, the expression of FMDV 3A and VP3 proteins were decreased in LRRC25-knockdown PK-15 cells compared with control cells (Fig. 8F). These results suggest that LRRC25 increases FMDV replication and growth.

### The 3A proteins of other picornaviruses also degrade G3BP1 by upregulating LRRC25.

**FIG 9:**
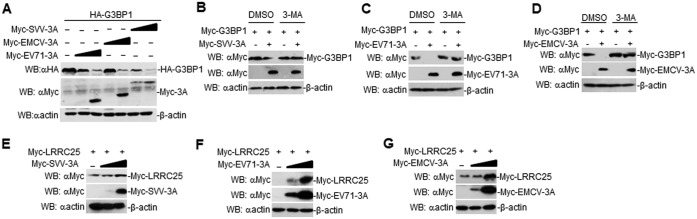
Picornaviral 3A degrades G3BP1 by the autophagy pathway. (A) Effects of SVV 3A, EMCV 3A, and EV71 3A on the expression of G3BP1. HEK293T cells were transfected with HA-G3BP1 (0.5 μg) and SVV 3A, EMCV 3A, or EV71 3A (0 μg, 0.2 μg, or 0.4 μg) for 24 h. Immunoblotting analyses were performed with the indicated antibodies. (B) SVV 3A degraded G3BP1 by the autophagy pathway. HEK293T cells were transfected with the indicated plasmids for 16 h. Cells were treated with dimethyl sulfoxide (DMSO) or 3-MA for 6 h. Immunoblotting analysis was performed with the indicated antibodies. (C) EV71 3A degraded G3BP1 by the autophagy pathway. The experiments were similarly performed as in B. (D) EMCV 3A degraded G3BP1 by the autophagy pathway. The experiments were similarly performed as in B. (E) Dose-dependent effects of SVV 3A on the expression of LRRC25 in HEK293T cells. HEK293T cells (2 × 10^5^) were transfected with the Myc-LRRC25 (0.5 μg) and Myc-SVV 3A plasmids (0 μg, 0.1 μg, or 0.2 μg) for 24 h. The cell lysates were analyzed by immunoblotting with anti-β-actin or anti-Myc antibodies. (F) Dose-dependent effects of EV71 3A on the expression of LRRC25 in HEK293T cells. The experiments were similarly performed as in F. (G) Dose-dependent effects of EMCV 3A on the expression of LRRC25 in HEK293T cells. The experiments were similarly performed as in F.

Because 3A proteins are orthologous and, thus, conserved among all picornaviruses, we determined whether 3A proteins from other picornaviruses could degrade G3BP1. In transient-transfection and Western blotting experiments, overexpression of 3A of Seneca Valley virus (SVV), enterovirus 71 (EV71), and encephalomyocarditis virus (EMCV) degraded G3BP1 in a dose-dependent manner (Fig. 9A). Furthermore, it was found that 3-MA blocked SVV 3A-, EV71 3A-, or EMCV 3A-mediated G3BP1 degradation (Fig. 9B to D). Additionally, 3A of SVV, EV71, and EMCV increased LRRC25 expression (Fig. 9E to G). These data suggest that the picornaviral 3A degrades G3BP1 by upregulating LRRC25 expression.

## DISCUSSION

G3BP1 is a multifunctional protein that participates in many physiological processes, such as SG assembly, immune response, arteriosclerosis, and tumor promotion (24, 26, 27). In this study, we found that FMDV 3A interacted with G3BP1. G3BP1 enhanced RIG-I and MAD5 expression, but FMDV 3A degraded G3BP1 and inhibited G3BP1-mediated RLH signaling by upregulating LRRC25 expression.

It has been reported that FMDV L^pro^ cleaves G3BP1 and G3BP2 and FMDV 3C^pro^ cleaves G3BP1, which inhibits SG formation (20, 21). FMDV L^pro^ and 3C^pro^ do not interact with G3BP1, suggesting that FMDV L^pro^ and 3C^pro^ cleave G3BP1 by other host proteins. FMDV L^pro^ degrades many host proteins, such as interferon regulatory factor 3/7 (IRF3/7) (28) and eukaryotic translation initiation factor 4GI (eIF4GI) (29). FMDV 3C^pro^ also degrades many host proteins, such as NF-κB essential modulator (NEMO) (4), eIF4GI (30), and Src-associated in mitosis 68 kDa (Sam68) (31). Previous reports also suggested that FMDV 3C^pro^ can degrade transforming growth factor-activated kinase-1 (TAK1), TAK1-binding protein 2 (TAB2), inhibitor of nuclear factor kappa-B kinase alpha (IKKα), IKKβ, and myeloid differentiation primary response gene 88 (MyD88) (unpublished data). In this study, we demonstrated that FMDV 3A interacts with G3BP1 and degrades G3BP1 by autophagy.

It has been reported that FMDV 3A inhibited RIG-I and MDA5 expression (7) and FMDV could be solely recognized by MDA5 (32), but the mechanism remains unclear. In addition, FMDV 2C inhibited FMDV replication by increasing IFN-β production (33). In the study, FMDV 3A increased FMDV replication and degraded G3BP1 by upregulating LRRC25 expression, which inhibited RIG-I and MDA5 expression.

G3BP1 is a key component and a commonly used marker of SG (19). FMDV 3C^pro^ and L^pro^ cleaved G3BP1 to inhibit SG formation (20, 21). It has been demonstrated that G3BP1 mediated autophagy pathways (34), but the mechanism remains unclear. In addition, G3BP1 interacts directly with the FMDV IRES and negatively regulates its translation (35). This finding prompted us to determine the mechanism by which G3BP1 may be regulated by FMDV proteins to propagate FMDV replication. In this study, porcine G3BP1 enhanced RLH signaling and FMDV 3A inhibited G3BP1-mediated RLH signaling by upregulating the expression of autophagy-related protein LRRC25. These findings suggest that FMDV or FMDV proteins have a connection with SGs (G3BP1), autophagy, and the RLH signaling pathway, but the mechanism needs to be further studied.

The 3A-deletion FMDV mutants (3A D93 and 3A D133) can alter the host range of FMDV (22), but the mechanism is unclear. An explanation for the above mechanism by natural immunity could not be reached in this study. First, both FMDV 3A D93 and FMDV 3A D133 inhibited RLH signaling and G3BP1-mediated RLH signaling as well as FMDV 3A. Second, the deletion mutants of FMDV also inhibited poly(I·C)-triggered RLH signaling as well as wild-type FMDV.

The FMDV capsid protein VP2 induces autophagy through interaction with heat shock protein beta-1 (HSPB1) and activation of the eukaryotic translation initiation factor 2 subunit alpha-activating transcription factor 4 (EIF2S1-ATF4) pathways (36). In this study, we found that FMDV 3A inhibited G3BP1 expression and G3BP1-mediated RLH signaling by upregulating the autophagy-related protein LRRC25. This study indicates that FMDV 3A mediates the autophagy pathway, but whether FMDV 3A directly or indirectly mediates the autophagy pathway needs to be further studied.

Consistent with previous studies, it was found that G3BP1 and G3BP1E284A inhibit FMDV replication and growth (21). We found that FMDV also inhibited the expression of G3BP1E284A, suggesting that other FMDV proteins mediated G3BP1 degradation. Finally, we confirmed that FMDV 3A degraded G3BP1. First, FMDV 3A degraded G3BP1 and G3BP1E284A in a dose-dependent manner in HEK293T cells. Second, FMDV 3A inhibited endogenous G3BP1 expression.

**FIG 10:**
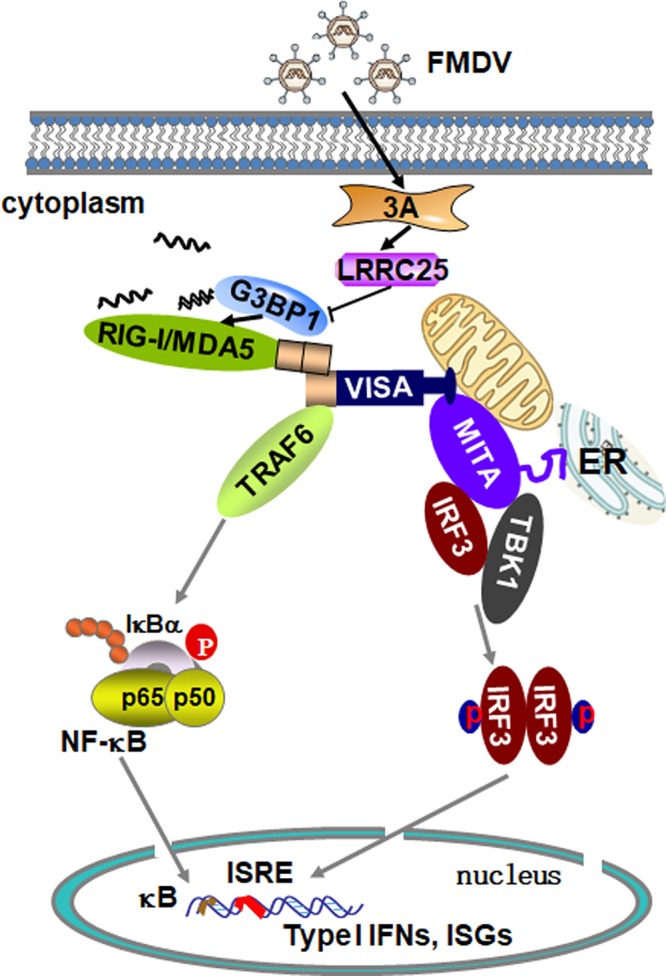
Working model of FMDV 3A regulation of the G3BP1-mediated RLH signaling pathway. Following FMDV infection, 3A, LRRC25, G3BP1, and RIG-I/MDA5 formed a complex. 3A inhibited the expression of G3BP1 by upregulating the expression of LRRC25, which resulted in a reduction in the expression of RIG-I and MDA5. Ultimately, this reduced the production of IFN-β.

Based on these findings, we propose a working model of FMDV 3A-mediated regulation of G3BP1 in innate response to FMDV. During FMDV infection, FMDV 3A, LCRR25, G3BP1, RIG-I, and MDA5 form a complex. Subsequently, FMDV 3A increases LRRC25 expression, which reduces G3BP1 expression to inhibit RIG-I and MDA5 expression (Fig. 10). These results provide important insights into the molecular mechanisms of FMDV 3A and G3BP1-mediated innate immune response and autophagy pathway for FMDV replication and growth.

## MATERIALS AND METHODS

### Reagents.

Mouse monoclonal antibodies against Flag, Flag-HRP, and Myc (Sigma); hemagglutinin (HA) (Covance); β-actin (Sigma); and rabbit polyclonal antibody against LRRC25 (Abcam) were purchased from the corresponding manufacturers. A mouse anti-VP3 and 3A polyclonal antibody was prepared using conventional methods. iQ SYBR green real-time supermix (Bio-Rad), Gammabind G plus Sepharose (Amersham Biosciences), and Moloney murine leukemia virus (M-MLV) reverse transcriptase (Invitrogen) were purchased from the indicated manufacturers. The human IFN-β ELISA kit was purchased from Pestka Biomedical Laboratorie. The porcine IFN-β ELISA kit was purchased from Solarbio Life Science.

### Viruses and cells.

HEK293T cells (ATCC) and PK-15 (ATCC) cells were grown in Dulbecco’s modified Eagle medium (DMEM) supplemented with 10% fetal bovine serum (FBS). The type-O FMDV and its deletion mutants were prepared in our laboratory and propagated in PK-15 cells, and the supernatants of infected cells were harvested and stored at –80°C for further studies. SeV was provided by Hongbing Shu (Wuhan University).

### Constructs.

Mammalian expression plasmids encoding VP0, VP1, VP2, 2B, 3B, 3C^pro^, 3D, L^pro^, and 3A; its mutants; and VP3 were constructed by PCR amplification of their cDNA from FMDV-infected PK-15 cells. Subsequently, they were cloned into cytomegalovirus (CMV) promoter-based vectors containing a Flag-tag or Myc-tag. The SVV 3A, EV71 3A, and EMCV 3A genes were synthesized and constructed into the plasmid vector containing Myc-tag. Mammalian expression plasmids for porcine HA-tagged or Myc-tagged G3BP1 and Myc-tagged LRRC25 were constructed by standard molecular biology techniques. Mammalian expression plasmids for the HA-tagged porcine G3BP1 mutant were constructed with standard molecular biology techniques. Mammalian expression plasmids encoding HA-RIG-I, Flag-RIG-I, HA-MDA5, and HA-VISA were described previously (25).

### TCID_50_ detection of FMDV.

The 50% tissue culture infective dose (TCID_50_) in the collected supernatants was determined by virus titration assay, as described previously (37). Briefly, baby hamster syrian kidney 21 (BHK-21) cells were resuspended in Dulbecco’s modified Eagle medium (DMEM) with 5% fetal calf serum (FBS) at a concentration of 1.5 × 10^6^ cells/ml, which was dispensed at 50 μl per well into 96-well flat-bottomed tissue culture plates. The plates were rocked to achieve uniform suspension thickness and were incubated at 37°C for 24 h to 36 h under 5% CO_2_ tension to attain 90% confluence. Serial 10-fold dilutions of virus stock prepared in FBS-less DMEM were added in 50-μl volumes to all wells. Plates were incubated at 37°C and 5% CO_2_ for 72 h, and the presence or absence of cytopathic effect (CPE) was then monitored. TCID_50_ was calculated by the Reed-Muench method.

### Transfection and reporter gene assays.

The HEK293T cells (∼1 × 10^5^) were seeded on 48-well plates and transfected the following day by standard calcium phosphate precipitation. In the same experiment, empty control plasmid was added to ensure that each transfection received the same amount of total DNA. To normalize for transfection efficiency, 10 ng of pRL-TK renilla luciferase reporter plasmid was added to each transfection. Luciferase assays were performed with a dual-specific luciferase assay kit (Promega). Firefly luciferase activities were measured and normalized to renilla luciferase activities.

### RNAi experiments.

Double-stranded oligonucleotides corresponding to the target sequences were cloned into the pSuper-retro RNAi plasmid. Small interfering RNAs corresponding to the same target sequences were purchased from GenePharma. The following sequences were targeted for porcine G3BP1 and LRRC25 cDNA, respectively: 5′-TGTCCATAGACTGCATCTGC-3′ and 5′-GGACGTGACAAACAACCCAC-3′.

### Coimmunoprecipitation and Western blotting.

For transient-transfection and coimmunoprecipitation experiments, HEK293T cells (1 × 10^6^) were transfected with the respective plasmids for 24 h and then were lysed in 1 ml of lysis buffer (20 mM Tris [pH 7.5] 150 mM NaCl, 1% Triton-X, 1 mM EDTA, 10 μg/ml aprotinin, 10 μg/ml leupeptin, and 1 mM phenylmethylsulfonyl fluoride). For each immunoprecipitation, a 0.4-ml aliquot of lysate was incubated with 0.2 μg of the indicated antibodies or control IgG and 25 μl of 1:1 slurry of Gammabind G plus Sepharose (Amersham Biosciences) for 2 h. The Sepharose beads were washed three times with 1 ml of lysis buffer containing 500 mM NaCl. The precipitates were analyzed by Western blotting as previously described (38).

For endogenous coimmunoprecipitation experiments, PK-15 cells (5 × 10^7^) were infected with FMDV (multiplicity of infection [MOI], 1.0) for the indicated time. Cells were then lysed in 5 ml lysis buffer and the lysate was incubated with 1 μl of the indicated antiserum or preimmune control serum. The subsequent procedures were carried out as described above.

### RT-PCR.

Total RNA was isolated from the cells using TRIzol reagent (TaKaRa) and subjected to RT-PCR analysis to measure mRNA expression. The mRNA levels of specific genes were normalized to levels of GAPDH mRNA. The human gene-specific primer sequences were as follows: 5′-GAGTCAACGGATTTGGTCGT-3′ (forward) and 5′-GACAAGCTTCCCGTTCTCAG-3′ (reverse) for *GAPDH*, 5′-TTGTTGAGAACCTCCTGGCT-3′ (forward) and 5′-TGACTATGGTCCAGGCACAG-3′ (reverse) for *IFNb*, 5′-GCCGCATCGCCGTCTCCTAC-3′ (forward) and 5′-CCTCAGCCCCCTCTGGGGTC-3′ (reverse) for *TNFa*, 5′-GAGAGTGATTGAGAGTGGACCAC-3′ (forward) and 5′-CACAACCCTCTGCACCCAGTTT-3′ (reverse) for *Il8*, 5′-GGCAGCCCTCGCTGTCATCC-3′ (forward) and 5′-GCAGCAGGGTGTGGTGTCCG-3′ (reverse) for *Rantes*, 5′-TCATCAGGTCAAGGATAGTC-3′ (forward) and 5′-CCACACTGTATTTGGTGTCTAGG-3′ (reverse) for *Isg56*, 5′-GGTGAGAAGAGATGTCTGAATCC-3′ (forward) and 5′-GTCCATCCTTGGAAGCACTGCA-3′ (reverse) for *Ip10*, and 5′-TTCTCCACAAGCGCCTTCGGTC-3′ (forward) and 5′-TCTGTGTGGGGCGGCTACATCT-3′ (reverse) for *Il6*. The porcine gene-specific primer sequences were as follows: 5′-ACATGGCCTCCAAGGAGTAAGA-3′ (forward) and 5′-GATCGAGTTGGGGCTGTGACT-3′ (reverse) for *GAPDH* and 5′-CACTGGCTGGAATGAAACCG-3′ (forward) and 5′-AATGGTCATGTCTCCCCTGG-3′ (reverse) for *IFNb*.

Viral RNA was extracted from FMDV-infected cells with TRIzol. Genomic copy numbers of FMDV were quantified by a previously described quantitative RT-PCR assay (39, 40).

### Statistical analysis.

The significance of differences between samples was assessed using an unpaired two-tailed Student’s *t* test. The variance was estimated by calculating the standard deviation (SD) and represented by error bars. All experiments were performed independently at least three times, with a representative experiment being shown.
